# Tunable Pyroresistive Behavior in Conductive Polymer Composites with a Secondary Elastomer Phase

**DOI:** 10.1021/acsaenm.6c00343

**Published:** 2026-07-13

**Authors:** Bijoy Das, Gordon Ip, Harshit Porwal, Jamie Evans, Mark Newton, Yi Liu, Dimitrios G. Papageorgiou, Han Zhang, Emiliano Bilotti

**Affiliations:** † School of Engineering and Materials Science, 4617 Queen Mary University of London, London E1 4NS, U.K.; ‡ LMK Thermosafe Ltd., 9-10 Moonhall Business Park, Helions Bumpstead Rd, Haverhill, Suffolk CB9 7AA, U.K.; § Department of Materials, Loughborough University, Loughborough LE11 3TU, U.K.; ∥ WMG, 2707 University of Warwick, Coventry CV4 7AL, U.K.; ⊥ Department of Aeronautics, Imperial College London, Exhibition Road, London SW7 2AZ, U.K.

**Keywords:** positive temperature coefficient, pyroresistive, conductive polymer composite, negative temperature coefficient, tunable

## Abstract

Positive-temperature-coefficient (PTC) polymer composites have emerged as a class of smart materials with notable interest and success, particularly in the field of overcurrent protection, self-regulating heating, and temperature sensing. However, a key problem with these composites is the negative temperate coefficient (NTC) effect, where past the peak PTC temperature, the electrical resistivity decreases due to the reagglomeration of the conductive filler. This opposing and unintended behavior compromises performance—which can be greatly hazardous in situ. Currently, physical (e.g., via irradiation) or chemical cross-linking of the composite polymer matrix remains the main method for negating NTC behavior, which substantially increases cost, reduces flexibility, and negates the possibility to recycle the material at the end of its life. Herein, we investigate a dispersed secondary-phase composite made of high-density polyethylene (HDPE), a high-temperature copolyester thermoplastic elastomer (TPE), and graphitic nanoplatelets (GNPs). It is demonstrated that the conductive polymer composite blend presents a unique and tunable pyroresistive response and a marked suppression of the NTC characteristic, even at very low content of the secondary polymer phase (2.5 wt % of TPE). Moreover, the formulation exhibits the ability to switch self-regulation temperature at specific ratios. The results are formulated through simple melt compounding without postprocessing, providing a cost-effective and safe solution to undesirable NTC properties and preserving the end-of-life option of thermo-mechanical recycling.

## Graphical abstract

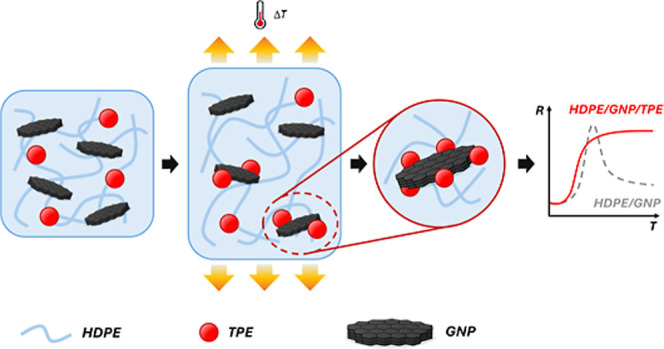

## 1 Introduction

In recent years, the positive temperature coefficient (PTC) effect in pyroresistive conductive polymer composites (CPCs) has garnered significant attention because of the increasing demand for smart materials. The PTC phenomenon refers to the sharp increase in electrical resistance with rising temperature within a specific temperature range, caused, in CPCs, by the disruption of a percolated conductive network created by fillers embedded in a polymer matrix. Such materials are promising candidates for self-regulating heating elements, overcurrent protection devices, and temperature sensors due to highly tunable electrical and thermal properties. In addition, the use of a polymer matrix allows for mechanical flexibility and ease of manufacturing of electronic elements and devices.^1,2^

In contrast to the PTC effect, the negative temperature coefficient (NTC) effect observed in CPCs entails a decrease in electrical resistance with increasing temperature and typically occurs past the PTC “peak” temperature. This occurs due to a reformation of the conductive network as the polymer viscosity decreases.^3,4^ This is typically undesirable and potentially unsafe in many applications. Several strategies have hence been explored in the field to suppress the NTC behavior. Cross-linking of the thermoplastic polymer matrix is the most common solution to eliminate the NTC effect. As often used to improve thermal properties and environmental stability, both chemical and irradiation cross-linking create covalent bonds between polymer chains, greatly reducing their ability to flow as temperature increases and stopping reagglomeration of the conductive network. Several studies have shown this in composite systems of polyethylene and carbon black (CB) and others.^5–10^ Tsao et al. showed that 80Mrads of γ-ray irradiation fully curtailed the NTC effect in HDPE/CB composites; however, it reduced the PTC intensity by 1 order of magnitude.^7^ Xie et al. found that the changes to PTC and NTC intensity were related to the thermal expansion properties of gel and sol regions in cross-linked composites and that the PTC intensity was increased by 2 orders of magnitude while the NTC intensity was reduced by 4 orders of magnitude in LDPE/CB composites treated with 400 kGy γ-rays.^5^ While effective, these methods greatly increase processing costs,^11^ reduce recyclability,^12^ and introduce potential safety concerns.^13^ As a result, alternative approaches are being investigated to suppress the NTC effect while maintaining material flexibility and cost-efficiency.

As NTC behavior is caused by the reagglomeration of filler particles in a low-viscosity (often liquid-phase) matrix, one such strategy to address NTC behavior is the modification of filler particles. Teng et al. treated barium titanate particles with a polydopamine coating and were able to suppress NTC behavior through improved filler–matrix adhesion to reduce filler mobility at elevated temperatures.^14^ Unfortunately, the modification of fillers in such a way is a largely unscalable process that requires several coating and rinsing steps and has only been achieved at lab scale, making it unsuitable for most industry applications.

Another strategy is by designing a mixed filler system. Carbon nanotubes can provide a level of mechanical and thermal reinforcement in a composite, as their high aspect ratio allows them to span across polymer phases, acting as a pseudo-crosslink, increasing viscosity and reducing filler mobility. Li et al. found that addition of small amounts (0.5–1 wt %) of multiwalled CNTs to carbon black-filled binary polymer formulations (UHMWPE/PP) suppressed NTC behavior compared to the CNT-less counterparts.^15^ However, the addition of another filler is again problematic due to the significant cost increase when involving CNTs and their large effect on electrical conductivity—the addition of even 1 wt % CNT greatly reduces the PTC intensity of the system, as the conductive network is too robust to break under thermal expansion. It is impossible to decouple the effect of room temperature conductivity, PTC intensity, and NTC suppression when using mixed fillers, and as such, it is typically not desirable to employ such a technique for industry.

Considered a far less expensive and more controllable option, several studies have investigated the use of binary polymer systems utilizing two immiscible polymers. Typically, the initial polymer will be chosen for exhibiting known PTC behavior, well reported for polymers such as LDPE,^5,16^ HDPE,^17–19^ and PP.^20–22^ The second polymer will then be chosen for improving an aspect of the composite, such as mechanical properties or processability. The interaction between the secondary polymer and the conductive network will almost always follow one of two behaviors. In many cases, the secondary polymer will have little or no impact on the overall PTC and NTC performance. Liu et al. blended three different thermoplastic elastomers (SEBS, EPR, and TPU) with a HDPE/GNP system and found that while some of the systems reported a reduction of room temperature conductivity (due to the dilution of the fillers in the secondary phase), no significant changes to the PTC switching temperature or overall PTC/NTC shape were observed.^23^ Other blends, such as carbon black-filled LDPE/EPDM, also reported no change in PTC temperature, intensity, or NTC behavior compared with a carbon black and LDPE system.^5,24^ The electrical behavior of these composites is indicative of a single filler network which resides in one polymer phase (or at the interphase) but is largely unaffected by the phase change and thermal expansion of the secondary polymer.

On the contrary, some binary polymer systems exhibit a double PTC effect, whereby two distinct PTC regions exist. This unique behavior arises from the coexistence of two different percolating networks within the composite material. As the temperature increases, the resistance of the CPC initially increases because of the breaking of the primary conductive pathway, marking the first PTC region. However, instead of transitioning into the undesirable NTC effect, the secondary volume expansion further affects the conductive pathway, leading to another sudden increase in the resistance and the onset of a second PTC region. If designed correctly, these two regions can be tuned to overlap, allowing the second PTC region to effectively negate the NTC behavior of the first network. This approach provides a promising strategy for stabilizing the resistive response of CPCs at elevated temperatures in a more tunable and scalable way than through filler modification or addition.

However, recurring limitations appear in double PTC systems reported in literature. First, changes in PTC behavior are only observed when the amount of additional, higher-temperature phase exceeds that of the initial polymer phase. Di et al. reported a very clear double PTC system with a HDPE/PP blend filled with carbon black (CB). This system exhibited two peaks, at 130 and 170 °C, corresponding to the melting temperatures of the two immiscible polymers. However, the effect was only possible at a very specific ratio of 40:60 HDPE/PP,^25^ with other formulations showing no effective change in NTC behavior. Feng et al. added tetrafluoroethylene–ethylene (ETFE) high-temperature copolymer to a HDPE/CB system and reported two PTC peaks at 130 and 200 °C. Again, any change in pyroresistive behavior was only visible at a ratio of 4:1 ETFE/HDPE—meaning the predominant and continuous phase was the exceptionally expensive ETFE.^3^ Zhang et al. added PVDF to a HDPE/CNF system, which again was able to show some improvement in NTC properties at a ratio of 4:1 PVDF/HDPE, with no useful change occurring with smaller proportions of PVDF.^26^

As these are often high-temperature polymers, the requirement to add them in such a large proportion will add a significant cost to the processing of such composites. Second, the conductivity achieved in many of the studies remains out of a useable range for practical applications, such as Joule heating.

Herein, we present a unique PTC system which exhibits a large suppression of the NTC effect when just 2.5 wt % (2 vol %) of a secondary thermoplastic copolyester elastomer (TPE) is added to a HDPE + 25 wt % GNP masterbatch. The composite maintains sufficient conductivity and PTC intensity for effective self-regulating Joule heating while demonstrating a strong suppression of the NTC effect up to temperatures of at least 170 °C. More distinctively, this system exhibits a continuous and tunable change in pyroresistive characteristics with addition of the secondary polymer, rather than an “all or nothing” change to two discrete double PTC peaks, as previously reported in literature. This presents a significant improvement upon common pyroresistive CPCs, without requiring large additions of the secondary high-temperature phase. The easy processability and good electrical conductivity give this material great potential, particularly for self-regulating heating and overcurrent protection.

## 2 Materials and Methods

### 2.1 Materials

Table 1 summarizes the polymers and fillers used, with relevant information listed. TPE polymer (Arnitel EM630-H, DSM-Firmenich) is an alternating block copolymer of 75% polybutylene terephthalate (PBT) and 25% polyoxytetramethylene (PTMO) polyether,^27^ selected for its high melting temperature and elastomeric properties. HDPE (Rigidex HD5218EA, INEOS Olefins & Polymers Europe) was selected for its high crystallinity and high PTC intensity, as reported in literature.^23,28–30^ Graphitic nanoplatelets (GNPs), (M-25 grade, XG Science) were selected for their large lateral size and good conductivity found previously.^16,23,30–32^ Particles had an average lateral size of ∼20 μm (from a previous measurement of 192 particles)^31^ and a thickness of 6–8 nm, as reported from the supplier.

**Table 1:** Materials Used with Relevant Datasheet Information

material	abbr.	supplier	trade name	composition	datasheet information
**matrix**					
PBT/polyether	TPE	DSM-Firmenich	Arnitel EM630-H	polybutylene terephthalate (PBT 75%)	density (kg·m^–3^): 1240
copolymer				polyoxytetramethylene (POTM 25%)	melt temp (°C): 212
					modulus (MPa): 275
					CTE (E-4/°C): 1.5
					crystallinity: 22.9
high-density polyethylene	HDPE	INEOS	Rigidex HD5218EA	polyethylene	density (kg·m^–3^): 952
					melt temp (°C): 130
					modulus (MPa): 1200
					CTE (E-4/°C): 2.2
					crystallinity: 58.9
**filler**					
graphene nanoplatelet	GNP	XG Science	M-25	carbon (>99.5%)	density (kg·m^–3^): 2200
					avg. lateral size (μm): 25
					avg. thickness (nm): 6–8
**solvents**					
ethylene glycol (anhydrous)	EG	Sigma-Aldrich	324558	HOCH_2_CH_2_OH	purity (%): 99.8
glycerol	-	Sigma-Aldrich	G5516	HOCH_2_CH(OH)CH_2_OH	purity (%): ≥ 99.0
di-ionised water	-	N/A	double distilled (type 1) water	H_2_O	resistivity (MΩ·cm): 18.2

### 2.2 Methods

#### 2.2.1 Sample Preparation

Initial formulations of unfilled HDPE and TPE were produced to investigate the morphology of the polymer blends. HDPE/TPE blends, in the weight ratios of 90/10, 70/30, 60/40, 50/50, 40/60, 30/70, and 10/90, were compounded in an Xplore MC15 Microcompounder at 260 °C and 50 rpm for 5 min. A Collin ZK25 Twin Screw Compounder was used to produce a HDPE/25 wt % GNP (15.8 vol %) masterbatch—herein referred to as MB. This filler percentage was selected for a good balance of conductivity and PTC intensity, as found in previous studies.^23,30^ Heating zones 1 (feeding) to 8 (die) were set to 185, 220, 220, 180, 180, 170, 160, and 145 °C, respectively. Screw speed was set at 180 rpm, and the polymer extrudate was fed immediately into a water bath before being pelletized with a Collin Teach Line pelletizer.

Conductive composite MB/TPE blends were made by compounding the previously created MB with various amounts of TPE polymer in the Xplore MC15 Micro compounder at 260 °C and 50 rpm for 5 min, in line with previous studies optimized in prior publications from the group but with consideration for the additional high-temperature polymer phase.^29,30,33^ Table 2 below details the percentage blends created.

**Table 2:** Formulations Created with Respective wt % and vol % of Each Component

	HDPE		TPE		GNP	
name	wt %	vol %	wt %	vol %	wt %	vol %
MB	75.0	87.4	0.0	0.0	25.0	12.6
MB/2.5	73.1	85.4	2.5	2.2	24.4	12.3
MB/7.5	69.4	81.5	7.5	6.8	23.1	11.8
MB/10	67.5	79.5	10.0	9.0	22.5	11.5
MB/20	60.0	71.4	20.0	18.3	20.0	10.3
MB/35	48.8	59.0	35.0	32.5	16.3	8.5
MB/50	37.5	46.1	50.0	47.2	12.5	6.7

Polymer pellets produced through compounding were then formed into several sample shapes through compression molding. Samples for conductivity, PTC, and Joule heating testing were produced to a dimension of 30 mm × 16 mm × 3 mm, with embedded copper mesh electrodes to minimize contact resistance. Films of neat HDPE and TPE were prepared for contact angle measurements. Dilatometer samples of neat polymers and unfilled polymer blends were pressed into 20 mm × 5 mm × 4 mm cuboids. All compression molding was carried out in a Collin P300E hot press at 270 °C and 60 bar of pressure for 5 min, followed by fast cooling under the same pressure.

#### 2.2.2 Characterization

##### 2.2.2.1 Scanning Electron Microscopy

Samples for scanning electron microscopy (SEM) were cryo-fractured after submerging specimens in liquid nitrogen for 10 min. An Inspect F (FEI) in primary electron mode was used to image fractured cross sections of gold sputter-coated samples under 10 keV.

##### 2.2.2.2 Thermogravimetric Analysis

Samples (10–20 mg) of pure polymer, pure GNP, and all CPCs were placed in platinum pans and loaded into a TGA 5500 thermogravimetric analyzer (TA Instruments). Samples were heated from room temperature to 600 °C at 10 °C/min in both a nitrogen atmosphere and air and held isothermally for 15 min. Decomposition characteristics were identified from the curve of the first derivative of the mass in temperature. GNP content was calculated from the residual mass of the samples at 500 °C in nitrogen after calibrating for the mass loss of the pure polymer and filler at the same temperature. Three samples were tested of each composition to calculate an average residual mass.

##### 2.2.2.3 Differential Scanning Calorimetry

Samples (5–15 mg) were placed in sealed aluminum pans for differential scanning calorimetry (DSC) (DSC25, TA Instruments) and subjected to a heat–cool–heat cycle between −80 and 300 °C at 5 °C/min. Heat flow data from the first cooling and second heating ramp were evaluated to find melting temperature (*T*_m_) and integrated with respect to time to find enthalpy of fusion (Δ*H*_m_). Degree of crystallinity (χ_c_) was calculated from Δ*H*_m_, using Equation S1 found in Section 1 of the Supporting Information. The enthalpy of fusion for 100% crystalline PBT (the crystalline phase of the TPE) was taken from literature as 145 J/g^34^ and for HDPE as 293 J/g.^35^ Three samples were tested of each composition.

##### 2.2.2.4 Contact Angle Measurement

A DSA100 Drop Shape Analyzer (Krüss Scientific) was used to measure the contact angle of 25 μL droplets on compression-molded flat polymer films. Surface energy calculations were made using contact angle measurements of deionized (DI) water, glycerol, and ethylene glycol (EG). Equation 1 from the Owens, Wendt, Rabel, and Kaelble (OWRK) model and eq 2 were then used to calculate surface and interfacial energies of HDPE, TPE, and GNP.^23,36^

1 $$\frac{\sigma_{L}\left(\cos\theta +1\right)}{\left(2\sqrt{\sigma_{L}^{D}}\right)}=\left(\sqrt{\sigma_{S}^{P}}\right)\frac{\sqrt{\sigma_{L}^{P}}}{\sqrt{\sigma_{L}^{D}}}+\sqrt{\sigma_{S}^{D}}$$

2 $$\gamma_{12}=\gamma_{1}+\gamma_{2}-2\left(\sqrt{\gamma_{1}^{D}\gamma_{2}^{D}}+\sqrt{\gamma_{1}^{P}\gamma_{2}^{P}}\right)$$

where σ_L_ and σ_S_ denote the surface energy of the liquid and solid, respectively. θ denotes the contact angle between the liquid droplet and polymer surface in radians. σ_1_, σ_2_, and σ_12_ denote the surface energies of component 1 and component 2 and the surface energy between components 1 and 2, respectively. Superscripts P and D denote the polar and dispersive contributions of these energies in both equations, respectively.

A wetting coefficient, eq 3 (based on a geometric mean equation), was then used to predict the location of the filler in the composite:^36^

3 $$\omega_{a}=\frac{\gamma_{filler,polymer1}-\gamma_{filler,polymer2}}{\gamma_{polymer1,2}}$$

where ω_*a*_ is the wetting coefficient, γ_filler,polymer_ is the interfacial energy between the filler and polymer (1 or 2), and γ_polymer1,2_ is the interfacial energy between polymers 1 and 2. These values are calculated from eq 2. A value of ω_a_ < 1 predicts the filler will reside in polymer 1. ω_a_ > 1 predicts the filler will reside in polymer 2, while 1>ω_a_ > −1 predicts the filler will migrate to the interface between the two polymers.

##### 2.2.2.5 Dilatometer

Compression-molded samples of pure polymers, blends, and CPCs were fitted into a Netzsch 402PC dilatometer to measure the coefficient of thermal expansion (CTE) of the pure polymer. Samples were heated from RT to up to 200 °C at 3 °C/min while measuring the length change of the sample. Three samples were tested with the average CTE reported.

##### 2.2.2.6 2-Probe Electrical Resistance Testing

A DC voltage from 0 to 50 V in 0.5 V increments was applied to each specimen with an Agilent HP 6614C power supply, while the electrical current was measured using a Pico-ammeter (Keithley 6485). The resulting current (*I*)–voltage (*V*) curves and sample dimensions were used to find the average electrical resistivity of 5 samples for each composition, with the early stage linear section of the curve (up to ∼10 V) used to calculate the resistivity.

##### 2.2.2.7 PTC Testing

Samples were heated in an oven from ambient to 220 °C at a rate of 3 °C/min, while the electrical resistance was measured with a combination of an Agilent (HP 6614C) power supply (1 V applied) and a picoammeter (Keithley 6485). The temperature was measured using a thermocouple affixed directly above the sample, with a TC-08 controller (Pico Technologies). Three samples were tested for each composition, with representative data shown in Section 3.

##### 2.2.2.8 Joule Heating Testing

Samples were connected to a variable DC voltage supply and set into an insulated box, with thermocouples affixed on top and bottom surfaces (with TC-08, a Pico Technologies controller). Voltages of 110 and 240 V were applied in turn by a Powerpro-300 DC power supply (Fisher Scientific International). Three samples were tested for each composition. Proving the PTC behavior of samples under Joule heating was done by providing a stepped voltage from 70 to 290 V at increments of 40 V (up to 230 V) and then 20 V. If heat losses are negligible, equations for input power *P* and heat output *Q* can be equated to each other and rewritten as eq 4:

$$Q=mC\Delta T/t\;\&\;P=\frac{V^{2}}{R}$$

4 $$V^{2}=R\cdot \Delta T\cdot C\cdot m$$

where *V*, *R*, *m*, and *C* denote the voltage, resistance, mass, and specific heat capacity of the sample, respectively, and Δ*T* the change in temperature. As *m* and *C* are constants during the Joule heating of a single sample, eq 4 shows that plotting temperature as a function of squared voltage should provide a linear (*R*) trend. However, under PTC behavior, this will change to show a clear difference between standard heating and self-regulation. As a steady-state scenario is approximated in experimentation, the time parameter Δ*T* can be ignored.

## 3 Results and Discussion

### 3.1 Morphology

The morphology of HDPE/TPE blends was first studied in samples without GNP to avoid the copious amounts of GNP flakes obscuring the boundaries of polymer phases. SEM images of each formulation are shown in Figure 1. It is visible that when TPE was added to HDPE, the blend exhibited an “island-in-the-sea” morphology of small globules/spheres, which increased in size as the ratio of TPE increased. At 90/10 HDPE/TPE, TPE globules were in a 1–5 μm range, while at 60/40 HDPE/TPE, TPE globules were in a 10–15 μm range, with some much larger globules too. At a 50/50 ratio, the morphology changed to co-continuous. The HDPE phase still contained globules of TPE; however, continuous regions of TPE were also present through the material.

**Figure 1:**
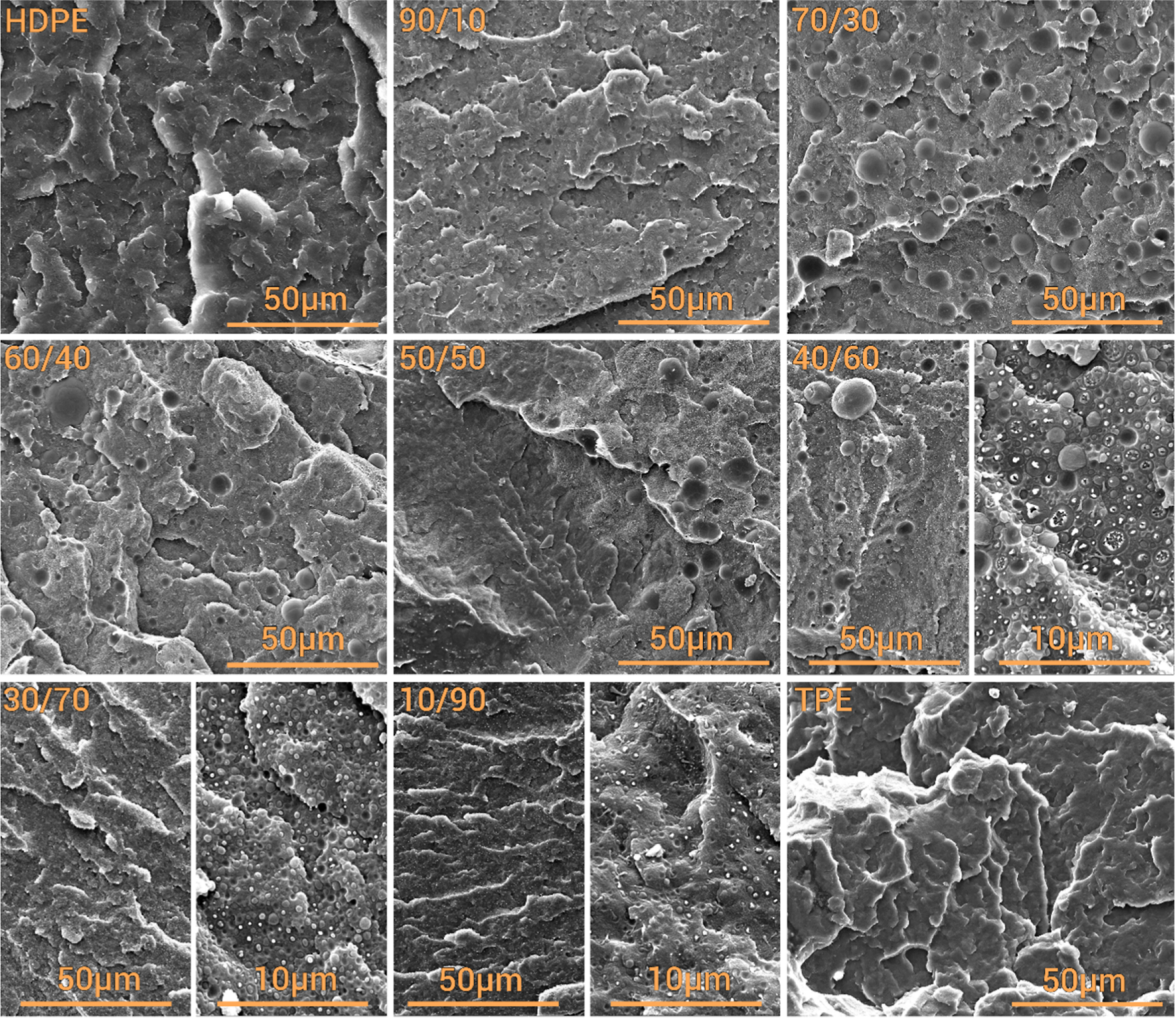
SEM micrograph of HDPE/TPE blends of (from top-left to bottom-right): pure HDPE, 90/10, 70/30, 60/40, 50/50, 40/60, 30/70, 10/90, and pure TPE. TPE existed in a droplet morphology in a continuous HDPE phase until 50/50, whereby a co-continuous phase could be seen. When TPE was the predominant phase, HDPE existed in droplets of decreasing size, existing in the nanoscale at 10/90 HDPE/TPE.

When the TPE phase was predominant (60/40 and onward), there was once again a change in the morphology. The composite reverted to an “island-in-the-sea” morphology with the phases reversed, with TPE as the continuous phase and globules of HDPE dispersed throughout. However, a key difference was the size of the HDPE regions, existing as microscale globules, with HDPE phases at 10/90 HDPE/TPE existing in the nanoscale. The morphology of the blends is asymmetric, with very small (nanoscale) structures visible in the predominantly TPE formulations.

### 3.2 Thermal Properties

Figure 2 shows the thermal properties of neat polymers, MB, and MB/TPE composites. DSC melting endotherms are shown in Figure 2A, with degree of crystallinity χ_c_ in Figure 2B. Pure HDPE exhibited an χ_c_ of 58.9%, as in previous literature.^37^ The addition of GNP to create the MB showed little effect on crystallinity, maintaining an χ_c_ of 57.5%. Our group previously reported TPE to have an χ_c_ of 22.9%.^31^ Herein, the TPE/MB blends showed a level of χ_c_ consistent with the proportion of the lower crystallinity TPE added, while the χ_c_ of the HDPE and TPE phases themselves remained constant. Visually, a melting endotherm could be seen at 10 wt % TPE; however, as reported in Table S1 in the Supporting Information, integration of the melting curve reported a small peak associated with TPE even at 2.5 wt % TPE. Blending TPE and the HDPE/GNP MB did not shift the melting temperatures of either polymer, with two distinct melting points at 130 and 216 °C for HDPE and TPE, respectively. Full values of melting/crystallization temperatures and χ_c_ can also be found in Table S1.

**Figure 2:**
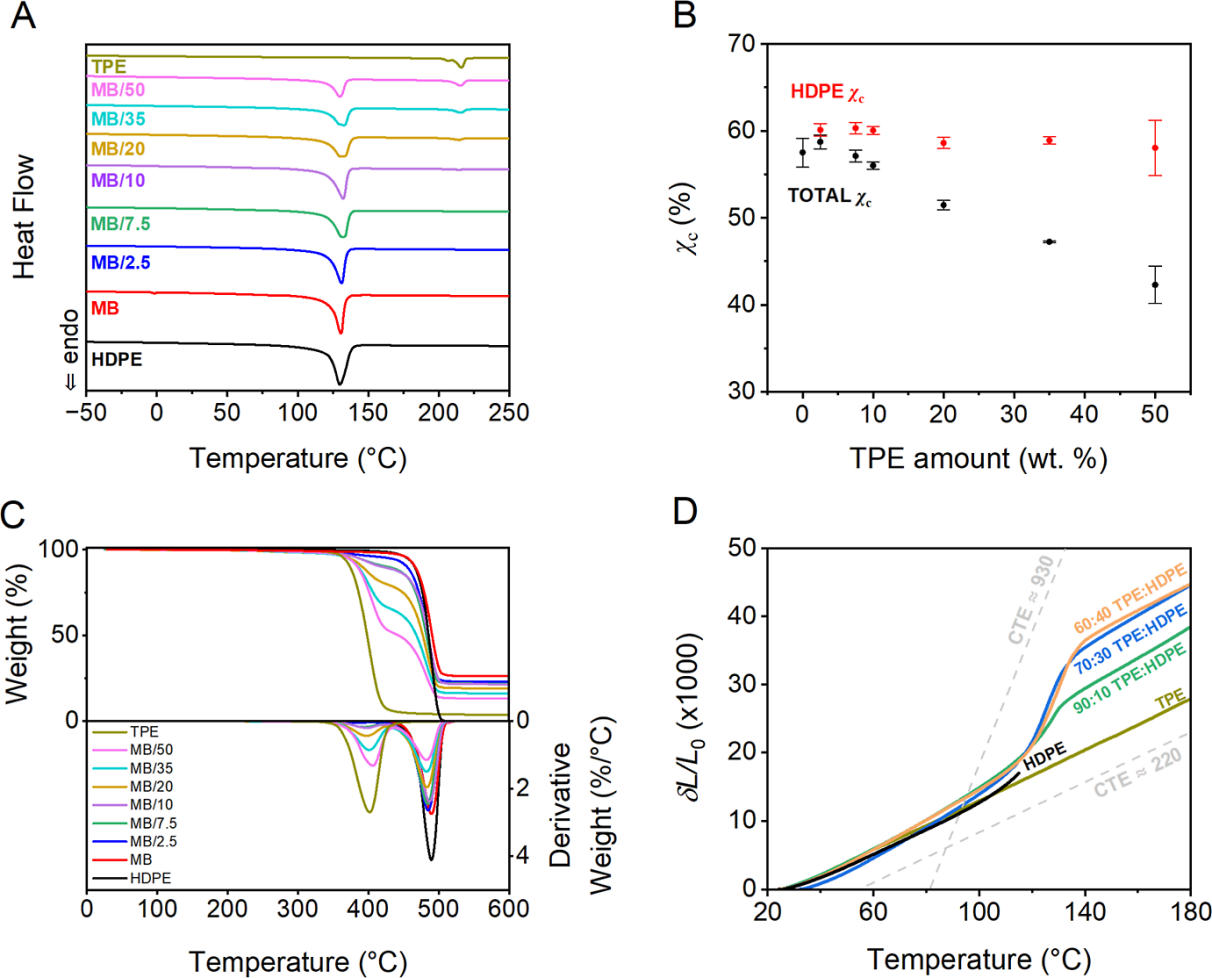
(A) Melting endotherms for neat polymers and all composites, showing two distinct melting points, respectively, for HDPE (130 °C) and TPE (216 °C). (B) χ_c_ of all loaded formulations. χ_c_ of the HDPE phase remained constant, while the total χ_c_ of the composite reduced as the TPE phase increased in percentage. (C) TGA data (with an inset derivative graph) showing a decomposition onset of 400 °C for TPE and 490 °C for HDPE. (D) Dilatometry data for HDPE, TPE, and unfilled blends. TPE and HDPE had a similar CTE of ≈ 200 μm/m°C, while the HDPE melting transition had a considerably greater average CTE of ≈ 930 μm/m °C, indicated by the gradient of the lines (gray dotted guidelines as a reference).

Figure 2C shows TGA data for all formulations. Blends showed two clear decomposition temperatures, respective to TPE (400 °C) and HDPE (490 °C), with a clear shift in the proportion of decomposition consistent with the proportion of the two polymers.

To better understand the expansion mechanism responsible for PTC behavior, the thermal expansion behavior of neat polymers and unfilled blends was investigated (Figure 2D). Measurements were limited past the melting point of HDPE in samples with greater than 50% HDPE content. Nevertheless, some clear observations could be made. While both neat HDPE and TPE exhibit a very similar linear thermal expansion coefficient of 210–220 μm/m °C, the presence of TPE allowed for the measurement of CTE at elevated temperatures past the melting point of HDPE. This was significant, as in both the dilatometry and PTC testing, the melting point of HDPE will act as the physical limit of measurement—past this point, the material has lost physical constitution, leading to NTC behavior. With TPE present, it could be measured that in the temperature range of 110–140 °C, the thermal expansion of the HDPE phase increased to between 500 and 1000 μm/m °C (dependent on HDPE percentage), indicated by the calculated gradient of the lines in Figure 2D. This is because the densely packed, organized molecular chains in the crystalline phase lose their structure during melting, converting to an amorphous structure which is far less dense—greatly increasing the volume of the material. In a HDPE/GNP system, this “melting expansion” of the CTE profile initiates the PTC behavior; however, loss of structural integrity past the HDPE melting temperature causes the onset of the NTC effect following any further heating. However, in TPE/HDPE blends, the increase in CTE during the HDPE melting process is bolstered as temperature increases by the continued linear expansion of the solid TPE phase. While the presence of TPE does not “increase” the thermal expansion of each respective phase, it allows for the “unlocking” of the full HDPE volume expansion during melting while still retaining suitable structural integrity due to the TPE, for both measuring in dilatometry and using for PTC behavior. Furthermore, the total amount of expansion will increase with the amount of HDPE in the composition, as the large expansion of melting material is the driver for the total volume increase. It could therefore be considered that formulations with the smallest amount of TPE to still retain structural rigidity would provide the greatest PTC intensity and continue to provide functional expansion (and PTC behavior) past the HDPE melting, provided that enough TPE is present to retain the material’s physical constitution. In turn, this should negate NTC behavior, as the material is still expanding with a viscosity high enough to inhibit filler reagglomeration.

### 3.3 Electrical Properties

A hypothesis was formed that a blend of HDPE and TPE could provide continued thermal expansion past the melting point of HDPE and thus more desirable pyroresistive performance. The greater the HDPE content, the larger the “melting expansion” and thus the PTC intensity would be, while the proportion of TPE phase would need to be large enough to provide suitable thermal reinforcement to maintain structural stability past the HDPE melting point. A blend with the minimum amount of TPE to retain the physical constitution of a specimen would provide the greatest PTC intensity while suppressing NTC behavior, as the sample would be able to make use of the HDPE volume expansion through the entire melt phase while being reinforced in both mechanical strength and expansion properties by the TPE past 130 °C, rather than the entire matrix existing as a melt state where GNP particles can reagglomerate.

This was tested through measurement of electrical properties for MB/TPE composites in Figure 3. In Figure 3A, a percolation threshold of 8 wt % (3.8 vol %) was measured for the HDP/GNP MB, corroborating previous studies.^23^ HDPE/25 wt % GNP had previously shown sufficient electrical conductivity and PTC intensity for use in self-regulating heating and thus showed promise for use as a masterbatch for blending with TPE. Addition of increasing amounts of TPE showed negligible reduction in conductivity even when up to 50 wt % TPE was added, suggesting there was little dilution of the GNP into the secondary phase. This was corroborated with SEM images from Figure 1, which show that TPE exists as globules before shifting to a co-continuous phase at 50 wt %. This behavior is indicative of “exclusion theory”, whereby GNP particles are constrained to a reduced volume by adding an exclusionary phase to the composite microstructure, thus strengthening the network, rather than being diluted through both phases.^38–41^ Moreover, in binary polymer systems, it is possible for GNP to reside—at least partially—at the interface between polymers, further strengthening the network in even smaller regions.^23^ From Figure 1, it was clear that in this system, the TPE globules exist in a size range smaller than GNP—suggesting that instead of filler particles residing *within* an interphase, the particles could exist in contact with smaller TPE globules, which would provide a level of thermal protection and reduce their mobility—while also being affected by the linear thermal expansion of TPE.

**Figure 3:**
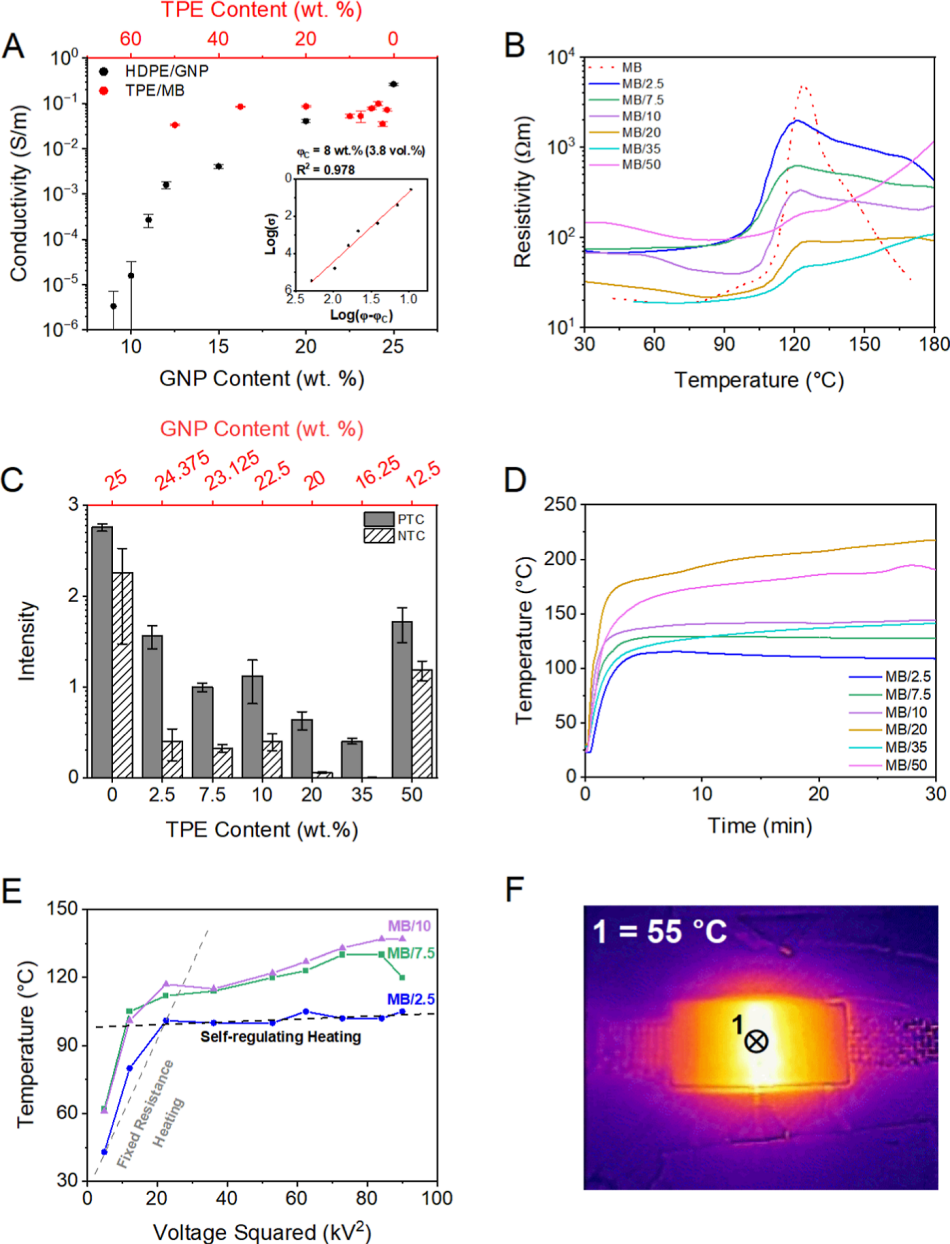
(A) Conductivity and percolation threshold of HDPE/GNP and MB/TPE composites, showing a percolation threshold of 8 wt % (3.8 vol %) and little change in conductivity as TPE was added. (B) PTC behavior of MB/TPE composites showing improved NTC characteristics with TPE addition, particularly at 2.5 wt % TPE. (C) PTC and NTC intensity of all formulations, quantifying PTC and NTC intensities. (D) Joule heating of all formulations at *V* = 240 V, showing steady-state heating in all but two formulations. (E) Stepped voltage squared test to show self-regulating heating compared to theoretical fixed resistance heating. (F) Representative thermal image of the sample under Joule heating at *V* = 240 V.

Evidence for the above can be seen in the PTC data in Figure 3B. The HDPE/GNP MB exhibited similar behavior to previous works, with a clear PTC effect at ∼130 °C with high intensity and a subsequent NTC effect.^23^ However, the addition of TPE substantially changed the pyroresistive behavior. Instead of a typical double PTC effect occurring at a certain blend ratio, even minor additions of TPE created an incremental “shift” in PTC behavior from a purely HDPE-governed switching point (∼130 °C) to that of a purely TPE/GNP system, with the PTC switching temperature of above 200 °C, as previously reported.^31^ This was unique to this system, as two-polymer systems reported in literature typically do not show any change in PTC behavior unless the higher-temperature phase is dominant/continuous.^3,25,26,28,42–44^ Several formulations (MB/20 and MB/35) exhibited almost no NTC effect at all. As little as 2.5 wt % of TPE added into the MB was sufficient to bring about significant improvements to NTC characteristics, quantified in Figure 3C—where PTC and NTC intensities are compared for all formulations. PTC intensity was measured as the minimum resistivity divided by the peak (maximum) resistivity, and NTC intensity was measured as the maximum resistivity divided by the reference resistance value at 170 °C. As the PTC peak for MB/50 shifted to 200 °C, an NTC temperature of 230 °C was chosen for this formulation. A marked improvement of NTC characteristics was observed with the addition of TPE, with the NTC intensity of MB/2.5 reduced by ∼2 orders of magnitude compared to MB, supporting the hypothesis above.

It can be noted that for some samples, a degree of thermal annealing was apparent, whereby conductivity increased in the early heating phase (up to 80 °C) of some samples. This effect has been documented in previous literature in the group,^45,46^ which, however, did not have any substantial effect on the overall PTC/NTC behavior changes.

The change in PTC behavior implies the movement of GNP with even small additions of TPE. The core principle of PTC behavior is the breaking of the conductive network under thermal expansion, and the NTC behavior seen in MB samples is the result of filler reagglomeration as the HDPE matrix is in a low-viscosity melt state. GNP solely residing in the HDPE phase would be unlikely to show any change in PTC/NTC behavior, as expansion of small local TPE globules would do little to break these pathways. The initial reduction in PTC intensity in all the blend systems, therefore, indicates that the conductive network is affected by both HDPE and TPE expansion—suggesting migration to an interphase. However, as per Figure 1, the size of TPE globules (<1–5 μm) is far smaller than the average GNP particle (∼20 μm)—suggesting that, rather, TPE globules will reside next to GNP particles. Such a morphology would first reduce PTC intensity as TPE content increases—evident in Figure 3C. However, it would also provide thermo-mechanical reinforcement which retained a high viscosity at temperatures past the HDPE melt, suppressing filler reagglomeration through physical blocking, while also providing solid-phase volume expansion throughout the temperature range. This can explain the reduction in NTC behavior, observed in formulations with just 2.5 wt % TPE in the composite. The reason for migration of GNP toward TPE globules (or vice versa) would likely be due to surface energy interactions, typical in binary polymer systems—investigated later in this work. Such behavior would (a) reduce the PTC intensity, as some of the volume surrounding the GNP is not expanding at the same rate as HDPE during its melting phase, and (b) interfere with GNP reagglomeration, suppressing NTC behavior—both seen in Figure 3B.

All GNP-loaded formulations were also tested for Joule heating, as shown in Figure 3D. Most formulations were able to provide steady-state heating to 110–120 °C; however, in the case of MB/20, the heating generated with 240 V could not be curtailed, and the temperature continued to increase until sample failure. This was due to the lack of PTC intensity, reported for all formulations in Figure 3B. MB/20 reported an *I*_PTC_ of less than 1 order of magnitude—not enough to provide a suitable “switch” effect and fully curtail current flow. In the case of MB/50, the PTC switching temperature had fully switched to that of the TPE phase and, as such, had a higher steady-state temperature approaching the 200 °C PTC switching point as reported in ref 31.

In this blend system, the lower crystallinity TPE polymer is providing a continuous thermal expansion of around 220 μm/m °C; however, the largest contribution to the volume expansion of the system during PTC behavior is the melting of the HDPE crystalline phase—during which the CTE increases to over 900 μm/m °C, as shown in Figure 2D. Here, HDPE content around the filler particles was insufficient to stop the current flow and regulate heating, resulting in a slow but constant temperature increase. Three chosen formulations were tested under stepped voltages from 70 to 300 V to verify self-regulation through the PTC effect, as described by eq 4. PTC behavior was verified in all three samples through the change in gradient, as a fixed resistance heater would provide a linear increase in temperature proportional to *V* ^2^, while a gradient change signifies a nonlinear resistance–temperature relationship in the material, as shown in Figure 3E.

Figure 3F shows a representative thermal image of a sample under Joule heating. As reported previously,^18^ the samples were hottest at the center, perpendicular to the flow of electrons. This is due to the comparative heat dissipation being greater at the edges of the sample (where electrodes and wires are connected) than the center, resulting in a temperature gradient, which in turn creates a proportional resistivity gradient. Under joule heating, the area of greatest resistance will heat the most, resulting in a thin line of greatest heating. The thickness of this line will depend on the specific heat dissipation characteristics, varying with the environment and sample geometry.

Overall, addition of TPE created unique and improved effects on the pyroresistive behavior of the HDPE/GNP MB. In particular, MB/2.5 showed vastly improved NTC characteristics, maintained suitable PTC intensity for self-regulating heating, and showed good repeatability under cyclic PTC (measured through stepped voltage Joule heating in Figure 3E). The proportional effect of TPE in this system was significantly greater than other PTC systems utilizing polymer blends in literature, which all require the higher-temperature polymer to be the predominant phase to have any effect on the pyroresistive behavior. Other formulations such as MB/20 and MB/35 completely neutralized NTC behavior, albeit with a reduction in PTC intensity—potentially more suitable for sensing applications over self-regulating heating. Not only was the amount of secondary polymer required to cause a change to the pyroresistive behavior significantly lower than reported previously, but it also exhibited an incremental and tunable behavior. A clear, cumulative shift in PTC behavior was observed with each greater addition of TPE, with MB/50 (TPE now the predominant phase) exhibiting both a PTC switching temperature and curve gradient similar to the previously reported TPE/GNP system.^31^ Both of these behaviors are unique and unreported in binary polymer PTC systems and suggest a new avenue of design for improving pyroresistive properties in a safe, simple, and cost-effective way.

### 3.4 Prediction of the GNP Location

The change in pyroresistive behavior strongly suggested that some level of interaction between the TPE and GNP network was occurring, as a completely noninteractive system would not show any change in PTC as the TPE secondary phase was added (such as the systems discussed in Section 1 ^23,24^ ). To better assess this interaction, a thermodynamic model was used to predict the preferred location of the GNP in a theoretical immiscible binary polymer blend system. In this model, GNP could preferentially reside in polymer 1 (HDPE), polymer 2 (TPE), or at the interphase, based on the minimization of interfacial surface energy. It should be noted that, in reality, the GNP location and overall composite morphology would also depend on the size of the phases with respect to each other.

Contact angle measurements of deionized (DI) water, glycerol, and ethylene glycol on flat surfaces of pure HDPE and TPE were used to determine their surface energies. Table 3 shows σ_L_^D^ and σ_L_^P^ for the solvents used, average contact angles θ, and the calculated elements of eq 1. Figure S1 (Supporting Information) was used to calculate σ_S_^D^and σ_S_^P^ for TPE and HDPE, which could then be used in eq 2 to find the wetting coefficient ω_a_ and predict which phase the GNP would reside in the composite—summarized in Table 4.

**Table 3:** Contact Angle Experimental Results and Calculated Values of Polar and Dispersive Elements of Respective Surface Energies

	LIQUID			HDPE			TPE		
	σ_L_^D^	σ_L_^P^	σ_L_	θ	$\frac{\sqrt{\sigma_{L}^{P}}}{\sqrt{\sigma_{L}^{D}}}$	$\frac{\sigma_{L}\left(\cos\theta +1\right)}{2\left(\sqrt{\sigma_{L}^{D}}\right)}$	θ	$\frac{\sqrt{\sigma_{L}^{P}}}{\sqrt{\sigma_{L}^{D}}}$	$\frac{\sigma_{L}\left(\cos\theta +1\right)}{2\left(\sqrt{\sigma_{L}^{D}}\right)}$
DI water	18.7	53.6	72.3	75	1.69	16.03	72	1.69	0.26
ethylene glycol	30.9	16.8	47.7	48.6	0.74	3.76	49.8	0.74	8.20
glycerol	37	26.4	63.4	81.5	0.85	10.32	70	0.85	8.63

**Table 4:** Wetting Coefficient and Prediction of the Filler Location in HDPE/TPE

	HDPE/GNP	TPE/GNP	HDPE/GNP
**γ**_12_	70.61	110.89	224.65
**ω**_***a***_	0.18		
filler location	interface		

In the case of a HDPE/TPE/GNP system, the model showed that the filler would preferentially reside in the interface between the two polymeric phases, as −1 < ω_a_ < 1. As we understand from the Figure 1 SEM images, TPE exists in small globules, which are much smaller than GNP particles, and as such would only partially wet the GNP particle network, initially residing only in the HDPE phase. This would (a) improve the thermo-mechanical stability of the network under thermal expansion past the melting point of HDPE (consistent with Figure 2D) and (b) act as a physical barrier to reconnection of the filler network—exhibited as a reduction of NTC behavior compared to HDPE/GNP (MB) samples (Figure 3). The reduction of PTC intensity with increasing TPE content, without affecting the electrical conductivity of the MB, is also indicative of TPE globules and GNP particles existing in local areas, together suggesting the filler is not diluted into the TPE phase (which would reduce conductivity); however, the presence of TPE reduces the proportion of the surrounding material around a filler particle that is melt-phase HDPE and therefore reduces the total local expansion around a given particle—resulting in a reduction in PTC intensity. Moreover, the loss of a PTC peak related to HDPE in the MB/50 composition suggests that when TPE is the predominant phase, it has created a GNP network that is mostly independent of the expansion of HDPE.

### 3.5 Verification of the GNP Location with SEM

The retention of a constant conductivity when TPE was added to the HDPE/GNP system (Figure 2A) suggested that GNP was not fully diluted into the HDPE and TPE phases (unlike the stepwise loss of conductivity seen in literature for HDPE/SEBS/GNP and HDPE/TPU/GNP systems^23^). However, the proportional reduction in PTC intensity with TPE addition (Figure 3C) indicated that TPE phases must be interacting or affecting the separation of the GNP network (only possible if they are existing next to each other). The prediction of GNP location based on interfacial energy also corroborated this, suggesting that in a theoretical system of HDPE and TPE, the GNP would preferentially reside at the interphase of the two polymers. As the TPE phases shown in Figure 1 were smaller than individual GNP particles, it was more likely that small TPE globules were partially “coating” GNP particles, acting as thermo-mechanical reinforcement and a physical barrier for GNP reagglomeration—reducing NTC behavior (Figure 3C).

In order to verify this, SEM images of the MB/TPE composites were taken to find decisive evidence that TPE existed next to GNP particles, where it would affect the electrical and thermal properties as seen in the previous data. Figure 4 shows MB/10, /20, /35, and /50 composites, with false color to highlight HDPE (green), GNP (blue), and TPE (red). In all formulations, regions of TPE could be seen next to the GNP particles in a continuous HDPE phase. This provides definitive evidence of the effect discussed above, whereby TPE polymer can provide excellent thermal stability and improved pyroresistive behavior due to its affinity with GNP particles, even when added in small wt. percentages.

**Figure 4:**
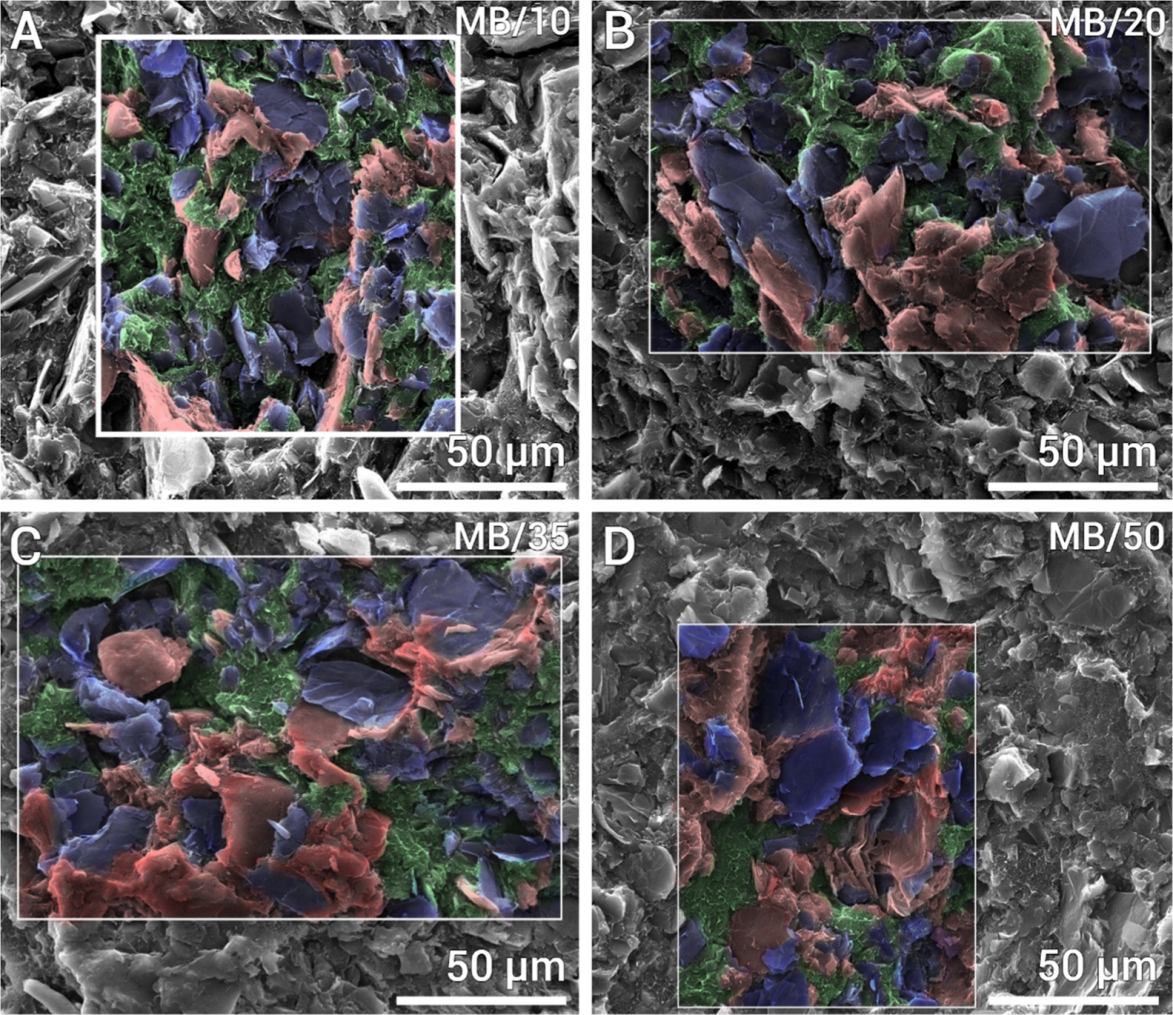
(A–D) SEM micrographs with false color to highlight HDPE (green), GNP (blue), and TPE (red) phases in (A) MB/10, (B) MB/20, (C) MB/35, and (D) MB/50 composites. In all imaged formulations, clear indications of TPE and GNP residing next to each other were visible, verifying the hypothesis discussed.

## 4 Conclusion

This study investigated the effect of a high-temperature thermoplastic elastomer as a minority secondary polymer phase on the PTC behavior of the well-studied HDPE/GNP system. The thermal and morphological characteristics of unfilled HDPE/TPE blends were first explored without the influence of GNP particles, showing that TPE existed in 1–5 μm spherical globules within a continuous HDPE phase in a 90/10 HDPE/TPE blend, increasing in globule size as TPE content increased (and vice versa). This provided a baseline of understanding about the morphology of the system.

Due to the innate link between PTC behavior and thermal expansion, dilatometry was used to investigate the volume expansion behavior of the blends. The use of a high-temperature secondary polymer provided suitable thermo-mechanical reinforcement for the HDPE phase, allowing for the full measurement of the HDPE *melting* expansion (between 110 and 140 °C), typically unmeasurable through dilatometry, where the CTE of HDPE rose from 210 μm/m °C to over 900 μm/m°C. The linear expansion of the TPE phase in this temperature range acted as a constant addition that could have a cumulative effect on the overall volume expansion, potentially improving NTC characteristics of a GNP-filled composite. The respective expansion properties of either polymer were not changed; however, the addition of a higher-temperature polymer allowed for (a) the measurement of HDPE expansion past its melting temperature and (b) (with regard to the GNP-filled composites) the HDPE melting expansion to fully affect the breaking of the conductive network, rather than this point being the limit of thermal stability and onset of NTC behavior. As predicted, this effect was greater with less TPE content, as more HDPE volume was present to undergo melting. However, the key dependency was the presence of enough TPE to ensure the samples remained physically stable past the HDPE melting point.

PTC data supported this hypothesis, reporting a considerable reduction of NTC behavior even at just 2.5 wt % TPE added to the HDPE/GNP masterbatch—a phenomenon never reported before in literature at such a low amount of secondary phase. Furthermore, formulations MB/20 and MB/30 TPE exhibited a complete suppression of NTC behavior, albeit with a reduction of PTC intensity of 2 orders of magnitude. This binary system was unique in its PTC behavior in that each small addition of secondary polymer provided a stepwise, tunable change to the PTC characteristics, completely unlike the “all-or-nothing” change seen in literature—which only occurs when the predominant phase is the higher-temperature polymer.

Surface energy calculations were used to model the 3-part system, which suggested a preferential location of GNP to the interphase of the two polymers, which was consistent with (a) lack of electrical conductivity change, (b) slight reduction in PTC intensity, and (c) reduction in NTC effect as TPE content was increased in the blend. Moreover, the respective size of TPE and GNP phases at lower TPE content heavily implied that rather than GNP residing at an interphase, TPE globules would exist on the surface of GNP particles, forming a barrier that retained high viscosity at elevated temperatures, providing improved thermal stability and preventing reagglomeration at the surface of GNP—ultimately reducing the NTC effect. Importantly, this was possible at a TPE content well below that reported in the literature of binary polymer PTC materials.

Overall, we propose that the addition of this high-temperature thermoplastic elastomer as the minority phase in a binary polymer blend could be an efficient strategy to tune the pyroresistive properties of CPCs, in particular in reducing the NTC effect and increasing the thermal stability without having to use cost and labor-intensive methods such as filler modifications or chemical/irradiation cross-linking.

## Supplementary Material



## Notes

Supplementary DSC data and table of crystallinity values of polymers and all produced blends and supplementary figure of the contact angle measurements, along with brief associated discussion (PDF)
