# Supplementary material for: Tunable Pyroresistive Behavior in Conductive Polymer Composites with a Secondary Elastomer Phase
Source: ACS Appl Eng Mater. 2026 Jul 13;4(7):3644–55. doi: 10.1021/acsaenm.6c00343 (PMC13411071; doi:10.1021/acsaenm.6c00343)
Supplement: Supplementary file 1 [file em6c00343_si_001.pdf]

Supporting Information:

Tuneable Pyroresistive Behaviour in Conductive Polymer Composites with a Secondary Elastomer Phase

Bijoy Das[1], Gordon Ip[1], Harshit Porwal[2], Jamie Evans[2], Mark Newton[2], Yi Liu[3], Dimitrios G. Papageorgiou, Han Zhang[4], Emiliano Bilotti[5]*

[1]School of Engineering and Materials Science, Queen Mary University of London, London, UK, E1 4NS.

[2] LMK Thermosafe Ltd., 9-10 Moonhall Business Park, Helions Bumpstead Rd, Haverhill, Suffolk, CB9 7AA, UK.

[3]Department of Materials, Loughborough University, Loughborough, LE11 3TU, UK.

[4]WMG, University of Warwick, Coventry, CV4 7AL, UK.

[5]Department of Aeronautics, Imperial College London, Exhibition Road, London, SW7 2AZ, UK.

**Corresponding Author email: e.bilotti@imperial.ac.uk*

Supporting Information

Section 1: DSC

Table S1 lists the melting temperature (T_M) taken as the peak of the melting endotherm, crystallisation temperature (T_C) taken as the peak of the crystallisation exotherm, and degree of crystallinity χ_c for all formulations. The χ_c was calculated using the below equation S1, for the TPE and HDPE segments seperately:

$$\chi_c = \frac{\Delta H_m}{(1 - w) \cdot \Delta H_m^0} \cdot 100\% \qquad \text{(S1)}$$

Where ΔH_m denotes the heat of fusion (calculated as the area under the respective HDPE and TPE melting endotherms), w denotes the weight fraction of filler content and ΔH_m^0 denotes the heat of fusion for a 100% crystal of the polymer, taken as 293 J/g and 145 J/g for HDPE and TPE respectively. It must be noted that as the filler was initially mixed into the HDPE phase, the filler content for the TPE phase was taken as 0.

From DSC data, two clear melting points of the HDPE and TPE phase can be seen at 130 °C and 215 °C respectively. With regards to crystallisation, all formulations with a blend of MB and TPE exhibited a crystallisation peak of around 200 °C, while neat TPE had a later onset of crystallisation at 179 °C, suggesting that in the presence of MB, the onset of TPE crystallisation is earlier during the cooling process due to nucleation sites in the presence of GNP. Earlier works surrounding TPE/GNP composites and literature in the field confirm the same results [1–3]. There was also a reduction in crystallinity of the HDPE phase as TPE content was increased. Literature suggests that this can occur due to a number of related mechanisms. The dilution effect of the secondary polymer makes it more difficult for crystallisable chains to find each other – reducing the efficiency of packing. A level of steric hinderance also occurs, hindering the spaces and movement of HDPE chains and disrupting the repeating, ordered

structuring of the crystalline phase. The secondary phase can also disrupt the normal heterogeneous nucleation sites that the host polymer uses to initiate crystallization, thereby slowing the crystallization rate and lowering the final fraction of crystalline material [4–6].

Table S1 – Melting (T_M) and Crystallisation (T_C) temperatures and degree of crystallinity χ_C for HDPE, TPE and all GNP-filled composites.

**Formulation**	**T_M HDPE** °C	**T_C HDPE** °C	**T_M TPE** °C	**T_C TPE** °C	**χ_C HDPE** %	**χ_C TPE** %	**χ_C Total** %
**HDPE**	129	116.3	N/A	N/A	58.9	N/A	58.9
**MB**	130.2	118.9	N/A	N/A	57.5	N/A	57.5
**MB/2.5**	131.0	116.9	N/A	N/A	58.6	0.1	58.7
**MB/7.5**	132.0	115.0	N/A	N/A	55.8	1.3	57.1
**MB/10**	131.7	115.1	215.0	201.4	54.3	1.9	56.2
**MB/20**	131.1	112.9	213.8	201.0	46.9	4.6	51.5
**MB/35**	132.9	112.6	215.4	200.5	38.3	8.9	47.3
**MB/50**	129.7	114.7	215.7	200.0	29.0	13.2	42.3
**TPE**	N/A	N/A	216.7	179.5	N/A	22.9	22.9

Contact Angle

Figure S1 uses the average contact angles θ of each solvent-polymer pairing to calculate the polar and dispersive components of both polymers. These values are then used in Equations 3 and 4 shown in Section 2 to predict the location of GNP particles, described in the main text.

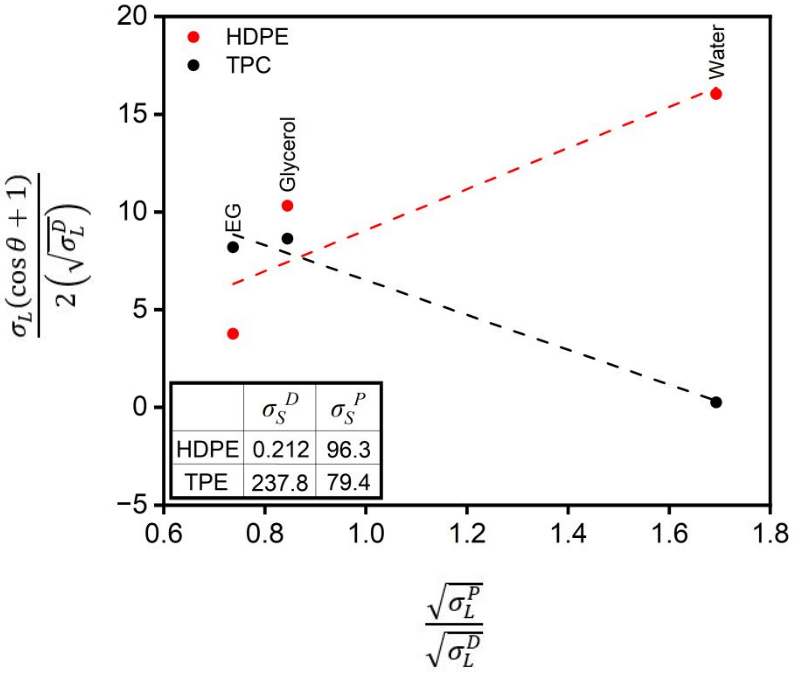

Figure S1 – Contact angle measurements plotted using Equation (2) to calculate σ_S^D and σ_S^P for TPE and HDPE pure polymers.

References

[1] *B. Das, X. Yu, Y. Wang, H. Porwal, J. Evans, M. Newton, D. Papageorgiou, H. Zhang, E. Bilotti, High temperature co-polyester thermoplastic elastomer nanocomposites for flexible self-regulating heating devices, Mater. Des. 242 (2024) 113000. https://doi.org/10.1016/J.MATDES.2024.113000.*

[2] *J.E.K. Schawe, Cooling rate dependence of the crystallinity at nonisothermal crystallization of polymers: A phenomenological model, J. Appl. Polym. Sci. 133 (2016). https://doi.org/10.1002/app.42977.*

[3] *A.J. Bourque, C.R. Locker, A.H. Tsou, M. Vadlamudi, Nucleation and mechanical enhancements in polyethylene-graphene nanoplate composites, Polymer (Guildf). 99 (2016). https://doi.org/10.1016/j.polymer.2016.07.025.*

[4] *G.K. Shambilova, R.M. Iskakov, A.S. Bukanova, F.B. Kairliyeva, A.S. Kalauova, M.S. Kuzin, E.M. Novikov, P.S. Gerasimenko, I.S. Makarov, I.Y. Skvortsov, Polypropylene Crystallinity Reduction through the Synergistic Effects of Cellulose and Silica Formed via Sol–Gel Synthesis, Polymers (Basel). 16 (2024). https://doi.org/10.3390/polym16202855.*

[5] *V. St-Onge, M. Cui, S. Rochon, J.C. Daigle, J.P. Claverie, Reducing crystallinity in solid polymer electrolytes for lithium-metal batteries via statistical copolymerization, Commun. Mater. 2 (2021). https://doi.org/10.1038/s43246-021-00187-2.*

[6] *H. Yang, J. Du, Crystallinity, Rheology, and Mechanical Properties of Low-/High-Molecular-Weight PLA Blended Systems, Molecules 29 (2024). https://doi.org/10.3390/molecules29010169.*
